# Enhanced Global-Brain Functional Connectivity in the Left Superior Frontal Gyrus as a Possible Endophenotype for Schizophrenia

**DOI:** 10.3389/fnins.2019.00145

**Published:** 2019-02-26

**Authors:** Yudan Ding, Yangpan Ou, Qinji Su, Pan Pan, Xiaoxiao Shan, Jindong Chen, Feng Liu, Zhikun Zhang, Jingping Zhao, Wenbin Guo

**Affiliations:** ^1^Department of Psychiatry, The Second Xiangya Hospital of Central South University, Changsha, China; ^2^Mental Health Center, The Second Affiliated Hospital of Guangxi Medical University, Nanning, China; ^3^Department of Radiology, Tianjin Medical University General Hospital, Tianjin, China

**Keywords:** schizophrenia, global-brain functional connectivity, functional magnetic resonance imaging, endophenotype, network

## Abstract

The notion of dysconnectivity in schizophrenia has been put forward for many years and results in substantial attempts to explore altered functional connectivity (FC) within different networks with inconsistent results. Clinical, demographical, and methodological heterogeneity may contribute to the inconsistency. Forty-four patients with first-episode, drug-naive schizophrenia, 42 unaffected siblings of schizophrenia patients and 44 healthy controls took part in this study. Global-brain FC (GFC) was employed to analyze the imaging data. Compared with healthy controls, patients with schizophrenia and unaffected siblings shared enhanced GFC in the left superior frontal gyrus (SFG). In addition, patients had increased GFC mainly in the thalamo-cortical network, including the bilateral thalamus, bilateral posterior cingulate cortex (PCC)/precuneus, left superior medial prefrontal cortex (MPFC), right angular gyrus, and right SFG/middle frontal gyrus and decreased GFC in the left ITG/cerebellum Crus I. No other altered GFC values were observed in the siblings group relative to the control group. Further ROC analysis showed that increased GFC in the left SFG could separate the patients or the siblings from the controls with acceptable sensitivities. Our findings suggest that increased GFC in the left SFG may serve as a potential endophenotype for schizophrenia.

## Introduction

Characterized by disturbances of perception (Yoon et al., 2008), cognition (Barch and Csernansky, 2007), emotion (Holt et al., 2011), and thought (Corlett et al., 2007), schizophrenia is a devastating and complex mental disorder, affecting adults as well as adolescence with highly heterogeneous and multifaceted clinical syndromes instead of a single disease entity (Yu et al., 2017). The diagnosis of schizophrenia is largely dependent on the psychiatrists’ evaluation and experience based on the comprehensive history records and laboratory examinations (Chin et al., 2018). In recent decades, great efforts have been made to identify reliable and objective biomarkers, such as electrophysiological (Turetsky et al., 2008; Smith et al., 2010; Edgar et al., 2012), neuropsychological (Smith et al., 2010; Edgar et al., 2012; Schulze-Rauschenbach et al., 2015), and neuroimaging indices (Edgar et al., 2012; Turner et al., 2012; Moran et al., 2013).

It has been postulated that schizophrenia is a neurodevelopmental disorder with abnormal neural connectivity of discrete brain networks and genetic and environmental factors may contribute to such dysconnectivity (Maynard et al., 2001; Karlsgodt et al., 2008). To date, substantial neuroimaging studies reveal structural and functional aberrations in many brain areas in schizophrenia or high risk populations, or both of them, including the prefrontal, cingulate, temporal, cerebellar, hippocampal, and thalamic regions (Rubinov and Bullmore, 2013; Thermenos et al., 2013; Bois et al., 2015; Chung and Cannon, 2015) within various brain networks such as the default-mode network (DMN) (Bluhm et al., 2007; Zhou et al., 2007; Ongur et al., 2010), cerebellar-cerebral networks (Konarski et al., 2005; Phillips et al., 2015), and thalamo-cortical networks (Andreasen et al., 1996; Jones, 1997; Swerdlow, 2010). Unaffected siblings of patients with schizophrenia, a subgroup of high risk subjects with approximately 50% of genetic burden (Pergola et al., 2017), have about a 10-fold increased risk to develop schizophrenia than general population (Chang et al., 2002). Unaffected siblings are free from confounding variables caused by environmental or disease-associated factors, and thus having an advantage to assess brain function with limited confounding factors. For example, disturbed resting-state FC has been observed in the first-degree relatives (Jang et al., 2011), which was predominantly altered in schizophrenia (Lynall et al., 2010; Skudlarski et al., 2010). Therefore, similar brain abnormalities shared by patients with schizophrenia and unaffected siblings can be regarded as potential endophenotypes for schizophrenia. Endophenotypes are some heritable and characteristic changes certainly present in patients but are possible to appear in unaffected relatives. They segregate with the disease within families and can be biochemical, neuroanatomical, cognitive, endocrine, or neurophysiological parameters (Gottesman and Gould, 2003; Bertolino and Blasi, 2009).

However, results from resting-state functional magnetic resonance imaging (fMRI) of abnormal intrinsic neural activity and/or functional connectivity (FC) across brain areas within those networks were inconsistent: increased FC (Zhou et al., 2007), decreased FC (Bluhm et al., 2007), or both (Ongur et al., 2010). One possible factor accounting for the mixed findings is that the majority of neuroimaging studies adopted either seed-based region-of-interest (ROI) analysis or independent component analysis (ICA), both of which are, to some extent, dependent on prior assumptions rather than employing a whole-brain examination (McKeown et al., 2003; Mannell et al., 2010; Joel et al., 2011). Therefore, it is possible to miss the most significantly altered regions which may indicate the core pathophysiology of schizophrenia.

Another reason may be that heterogeneous samples with different illness duration and medication history have biased the findings. Results from some longitudinal MRI studies in patients with chronic schizophrenia have showed accelerated gray matter loss over time and such progressive structural alterations were more remarkable at the initial stage of illness (Yoshida et al., 2009; Chiapponi et al., 2013;Schnack et al., 2016). As for resting-state fMRI studies, researchers have revealed reduced FC within the executive control network (ECN), DMN and dorsal attention network (DAN) in medicated patients (Woodward et al., 2011), whereas no changes were found within the ECN network in first-episode, drug-naive patients with schizophrenia (Lui et al., 2009). Therefore, it is essential to recruit first-episode, drug-naive patients with schizophrenia to explore the intact connectivity of these networks.

In the present study, we aimed to explore global-brain FC (GFC) differences by comparing a group of first-episode, drug-naive patients with schizophrenia and unaffected siblings with healthy controls employing the voxel-wise model-free GFC method, which had been described in details in our previous study (Cui et al., 2018). Apart from the seed-based ROI method and ICA method, GFC is another method of functional connectome which consists of FC of anatomically different brain areas (Craddock et al., 2013). Unlike the ROI and ICA methods, GFC is not biased by *a priori* specification of brain areas like ROI and spares from controversial views on the number of components in the ICA method (Kelly et al., 2012). Thus, the GFC method was preferable in our study. Based on the dysconnectivity hypothesis of schizophrenia and aforementioned studies, we hypothesized that patients with schizophrenia would reveal abnormal GFC in brain regions pertain to certain networks especially the DMN and thalamo-cortical circuit. Another hypothesis was that disrupted GFC could serve as an endophenotype shared by patients with schizophrenia and unaffected siblings. In addition, receiver operating characteristic (ROC) curve was conducted to differentiate the patients and unaffected siblings from the controls. Finally, we also examined correlations between disrupted GFC and clinical variables such as illness duration and symptom severity assessed by Positive and Negative Syndrome Scale (PANSS).

## Materials and Methods

### Participants

Forty-six patients with first-episode, drug-naive patients with schizophrenia, 46 non-affected siblings of patients with schizophrenia and 46 healthy controls took part in this study. All subjects were right handed, and aged from 18 to 37 years with more than 6 years of formal education. Handedness was determined by the Annett Hand Preference Questionnaire (Dragovic and Hammond, 2007). The study was in accordance with the Helsinki Declaration and approved by the local ethics committees of the Second Affiliated Hospital of Guangxi Medical University. All participants signed their written informed consent.

The included patients and siblings were recruited from the Mental Health Center, the Second Affiliated Hospital of Guangxi Medical University in China, and the controls were recruited from the local community. The diagnosis of schizophrenia was made by two research psychiatrists (W.G. and Z.Z.) according to the Structured Clinical Interview of the Diagnostic and Statistical Manual of Mental Disorders-IV (DSM-IV) criteria, patient edition, whereas non-patient version was used for unaffected siblings and healthy controls to rule out any psychiatric conditions. No antipsychotic medications or other psychotropic agents were treated to the patients, and PANSS total scores referring symptom severity of them was more than 70 at baseline. All participants did a series of routine physical examinations including systems review and laboratory tests to exclude any significant medical conditions and shared the same exclusion criteria: neurological disorders or history of brain injury, history of nicotine dependence, alcohol or other substance dependence, or any contraindications to MRI scan. In addition, potential controls who had a first-degree relative diagnosed with psychiatric disorders were also excluded.

### Imaging Acquisition and Preprocessing

Scanning was performed on a Siemens 3.0 T scanner. Participants with soft earplugs and foam, which could reduce scanner noise and head movement, were informed to lay still and remain awake with their eyes closed. After scanning, all subjects were asked some questions to claim that they did not fall asleep during the scanning. The images were acquired with a gradient-echo echo-planar imaging (EPI) sequence using the following parameters: repetition time/echo time (TR/TE) = 2000 ms/30 ms, 30 slices, 64 × 64 matrix, 90° flip angle, 24 cm field of view, 4 mm slice thickness, 0.4 mm slice gap, and 250 volumes lasting for 500 s.

Software DPABI was used to preprocess the imaging data (Yan et al., 2016). After slice timing and head motion correction, participants with over 2 mm maximal translation and 2° maximal rotation were excluded. Several covariates, including Friston-24 head motion parameters acquired through rigid body correction (de Kwaasteniet et al., 2013), signal from a ventricular region of interest, and signal from a region centered in the white matter, were removed. In addition, we applied mean frame-wise displacement (FD) according to a formula described previously (Liu et al., 2008; Power et al., 2012) to address the residual effects of motion as a covariate in group analyses. The global signal was not removed since it is still a controversial practice in the resting-state fMRI field (Hahamy et al., 2014). Then, we normalized the data to conventional EPI template in the Montreal Neurological Institute (MNI) space at a 3 mm × 3 mm × 3 mm resolution. Finally, the images were bandpass-filtered (0.01–0.08 Hz) and linearly detrended following spatially smoothed with a 4 mm full-width at half-maximum Gaussian kernel.

### GFC Analysis

Voxel-wise GFC method, defined as FC between a selected voxel and all other voxels in a given gray matter mask, was used to create voxel-to-voxel maps by composing GFC values of all voxels for each subject. SPM8 in Matlab (Liu et al., 2015) was used to generate the gray matter mask by setting the threshold at probability > 0.2. According to Yan and colleagues (Chao-Gan and Yu-Feng, 2010), a threshold of 0.2 was used to create a gray matter mask in this study, which indicated that voxels with the probability > 0.2 would be classified as gray matter. The GFC was computed as:

$$GFC_{\text{a}}=\sum_{\text{b=1}}^{\text{n}}\frac{r(T_{\text{a}},\;T_{\text{b}})}{n-1}$$

Where, Pearson’s correlation coefficient (r) was calculated at the given voxels a and b for *T_s_*, a pair of time series, followed by Fisher r-to-z transformation (Cui et al., 2018) and the GFC of a voxel was the coefficient of this voxel with all other voxels in the mask.

### Statistical Analysis

When appropriate, demographical data including age, sex, and years of education and clinical data were compared by using Chi-square test and analysis of variance (ANOVA).

After performing analysis of covariance (ANCOVA), *post hoc t*-tests were carried out to compare group differences among patients with schizophrenia, unaffected siblings, and controls. Age and the mean FD were applied as covariates in the ANCOVA and *post hoc t*-tests. The results were corrected by the Gaussian random field (GRF) theory at *p* < 0.05 (voxel significance: *p* < 0.001, cluster significance: *p* < 0.05).

After identifying brain regions with abnormal GFC values showing significant differences by group comparisons, the mean GFC values were extracted from these regions for further ROC curves analysis, which was used to examine whether these regions could discriminate patients with schizophrenia or unaffected siblings from healthy controls as reliable markers.

Linear correlation analyses were performed between abnormal GFC and clinical variables in PANSS scores and illness duration in the patient group (*p* < 0.05). The Bonferroni correction was used to limit type I error.

## Results

### Demographical and Clinical Characteristics

Two patients, 4 siblings, and 2 healthy controls were excluded due to excessive head motion. Therefore, the final analysis enrolled 44 patients, 42 non-affected siblings, and 44 healthy controls. The three groups had no significant differences in age, sex, education level, and FD values (see Table 1). The mean illness duration of the patients was 22.34 ± 7.01 months, and the mean PANSS total score was 90.70 ± 11.17.

**Table 1:** Baseline demographic and clinical characteristics of the study participants.

	Patients (*n* = 44)	Siblings (*n* = 42)	Controls (*n* = 44)	*p*-value
Gender (male/female)	28/16	28/14	23/21	0.35
Age (years)	23.45 ± 4.24	23.57 ± 3.62	23.55 ± 2.58	0.99
Education (years)	11.11 ± 2.46	12.13 ± 2.24	11.30 ± 1.67	0.11
FD (mm)	0.03 ± 0.03	0.03 ± 0.01	0.03 ± 0.02	0.34
Illness duration (months)	22.34 ± 7.01			
				
**PANSS**				
Positive symptom score	22.48 ± 5.37			
Negative symptom score	22.50 ± 6.38			
General symptom score	45.73 ± 6.97			
Total score	90.70 ± 11.17			

*FD, framewise displacement; PANSS, the Positive and Negative Syndrome Scale; Values are expressed as mean ± SD.*

### Group Differences in the GFC Values

**FIGURE 1:**
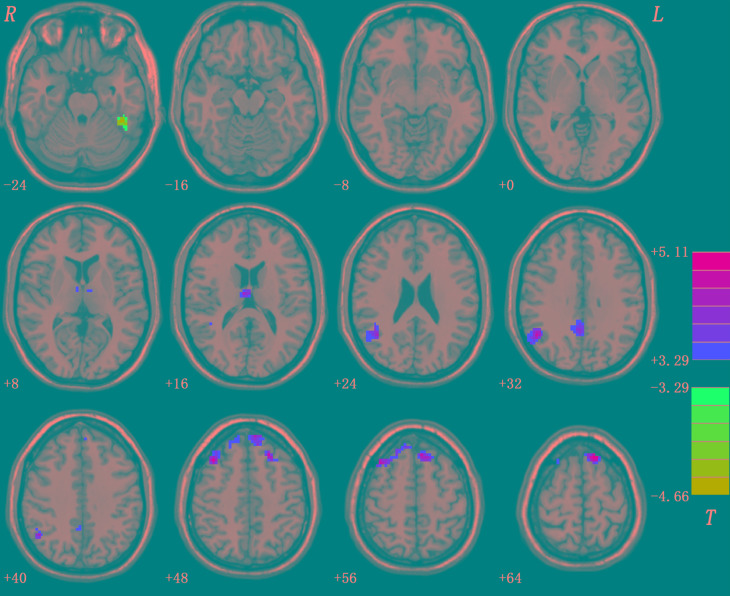
Abnormal GFC in patients with schizophrenia relative to healthy controls. GFC, global-brain functional connectivity.

**FIGURE 2:**
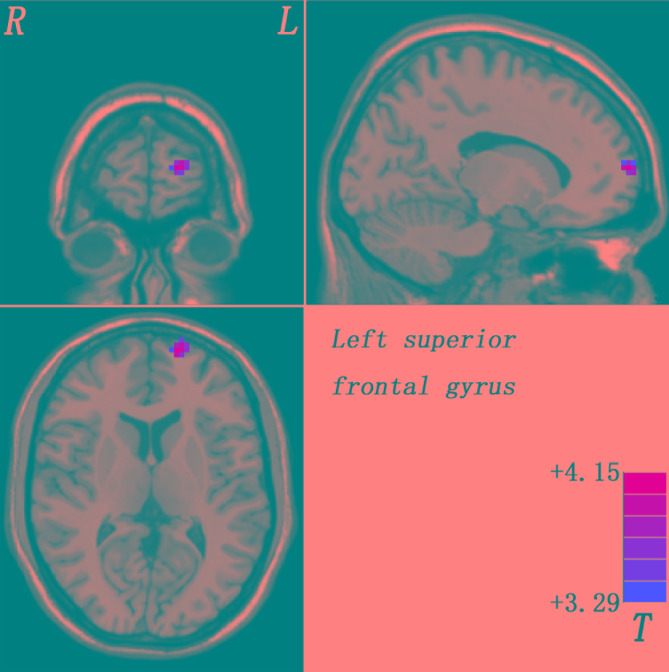
Enhanced GFC in the left SFG in the siblings compared to the controls. GFC, global-brain functional connectivity; SFG, superior frontal gyrus.

Compared with healthy controls, patients with schizophrenia and unaffected siblings shared enhanced GFC in the left superior frontal gyrus (SFG). In addition, as showed in Table 2 and Figure 1, the patient group had increased GFC in other areas such as the bilateral PCC/precuneus, and decreased GFC in the left ITG/cerebellum Crus I relative to the control group. No other altered GFC values were observed in the siblings group relative to the control group (Table 2 and Figure 2).

**Table 2:** Baseline group comparison in levels of GFC across groups.

Cluster location	Peak (MNI)			Number of voxels	*T*-value
	x	y	z		
**Patients vs. Controls**					
Left ITG/cerebellum Crus I	-45	-42	-24	55	-4.6571
Bilateral thalamus	6	-12	15	50	4.2670
Right angular gyrus	51	-57	33	139	4.6931
Bilateral PCC/precuneus	3	-54	33	67	4.0364
Left superior MPFC	-9	54	45	66	4.4945
Right superior frontal gyrus/middle frontal gyrus	39	24	48	150	5.0740
Left superior frontal gyrus	-9	27	60	113	5.1110
					
**Siblings vs. Controls**					
Left superior frontal gyrus	-15	66	9	28	4.1515

*GFC, global-brain functional connectivity; ITG, inferior temporal gyrus; MPFC, medial prefrontal cortex; PCC, posterior cingulate cortex.*

### Correlation Results

After the Bonferroni correction (*p* > 0.05/7 = 0.007 for abnormal GFC values in the seven brain regions), no significant correlations were found between GFC values and clinical variables in the patients.

### ROC Results

**FIGURE 3:**
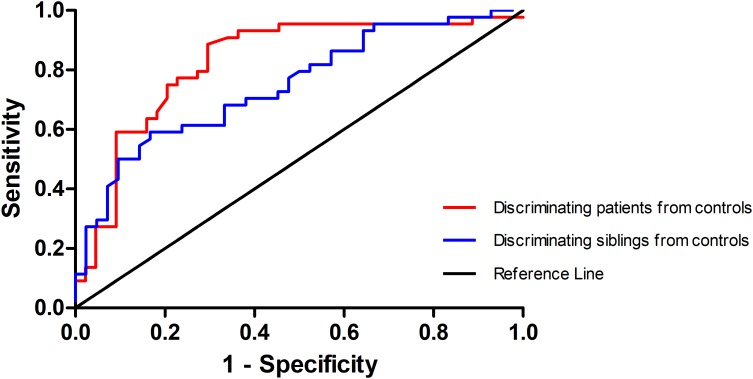
Receiver operating characteristic (ROC) curve of separating the patients and the siblings from the controls by using the GFC values in the left SFG. GFC, global-brain functional connectivity; SFG, superior frontal gyrus.

Since the left SFG exhibited increased GFC in both the patients and the siblings, it might be considered as a marker to separate the patients or the siblings from the controls. To examine this potential, ROC analysis was conducted. As shown in Figure 3, to discriminate the patients or the siblings from the controls, the areas under the curve of the left SFG were 0.829 or 0.748, respectively. Further diagnostic analysis showed that the sensitivity and specificity to separate the patients or siblings from the controls were 70.45 or 85.71%, and 90.91 or 56.82%, respectively.

## Discussion

In the present study, we first tested abnormalities of voxel-wise brain-wide FC in first-episode, drug-naive patients with schizophrenia and non-affected siblings using the GFC analysis. The key finding was that the patients and the siblings shared enhanced GFC in the left SFG relative to the controls. Further ROC analysis showed that the GFC value in this area might serve as a marker with a relatively high sensitivity to discriminate the patients or the siblings from the controls. Compared to healthy controls, patients with schizophrenia showed disturbed GFC mainly in the thalamo-cortical network.

There are two important features of our study. First, we explored FC abnormalities in patients with schizophrenia in an unbiased way using the voxel-wise brain-wide method. To date, not a unanimous pattern of brain functional anomalies pertaining to schizophrenia has converged among researchers, though these studies have indicated importance of abnormalities in certain brain circuits. The reason may be that many previous studies in this field focused on some predefined brain areas using approaches based on ROI (Guo et al., 2015a). It is conceivable that different studies obtained different results by selecting different ROIs. Additionally, it is possible that the most important brain regions relating to the core pathological changes in schizophrenia were never covered in some studies. On the contrary, the GFC method used in our study investigated the FC abnormalities in a voxel-wise brain-wide and more importantly, an unbiased way.

The second important feature is the sample groups recruited in this study. First-episode, drug-naive patients with schizophrenia were recruited to explore the intact connectivity of these networks in the present study. Except patients with schizophrenia, unaffected siblings were also enrolled in the study. Taking into account that schizophrenia is a highly heritable and complex disorder, unaffected siblings of schizophrenia patients who share remarkable genetic backgrounds with the patients are at a high-risk state to develop the disease (Jang et al., 2011). In order to have a more comprehensive insight into the neural underpinnings of schizophrenia, it is essential to investigate this group of people without interference of clinical and treatment matters. In addition, with more efforts putting into the effective treatment that could improve the clinical outcomes of patients with schizophrenia considerably, earlier identification and intervention are pushed to an urgent place (Chang et al., 2016). Investigating the vulnerability state and initial period of schizophrenia are help to address this issue.

The left SFG, involving in the impaired attention and cognitive domains (Wolf et al., 2008) including perception, working memory (Jenkins et al., 2018), and active imagery (Qiu et al., 2018), is one of the most consistently explored regions that may be a key hub in the pathophysiological processes of schizophrenia. In present study, increased GFC of the left SFG was found both in patients with schizophrenia and unaffected siblings and further ROC analysis exhibited that the GFC values of this region might be applied as a potential marker to differentiate the patients as well as the siblings from the controls with relatively high sensitivity. However, no correlations were found between the GFC value in this area and symptom severity or illness duration, which was somewhat out of our expectations. We supposed that the enhanced FC might be a trait alteration for schizophrenia independently of symptom severity and illness duration. The relatively small sample size was also a confounding factor. In addition, consistent with our results, many previous resting-state fMRI studies recorded no correlation between abnormal FC and clinical variables in patients with schizophrenia (Guo et al., 2015a). Actually, some researchers have reported a similar pattern of cognitive deficits between patients with schizophrenia and the first-degree relatives, including working memory, set shifting, and prepotent response (Johnstone et al., 2002; Brewer et al., 2005; Snitz et al., 2006). Similarly, a M100 magnetoencephalography study found greater left SFG M100 activity in not only patients with schizophrenia but also unaffected relatives (Chen et al., 2018). This shared auditory encoding abnormality indicated a compensatory adjustment by overactivating dorsal auditory pathway (Chen et al., 2013) and could also be regarded as a potential endophenotype.

The thalamus, associated with many brain functions such as cognitive and attention control (Carlesimo et al., 2011; Schmitt et al., 2017), goal-directed mental operation (Doucet et al., 2018), and experience and expression of emotion (Frodl et al., 2002), is a complex structure. Several neurobiological studies have postulated that the pathophysiology of schizophrenia involves abnormal functional interactions between the cortex and thalamus, the subcortical structure (Cheng et al., 2015). Our result of increased GFC in bilateral thalamus was consistent with previous studies, which found increased connectivity between thalamus and motor and somatosensory cortical areas (Woodward et al., 2012). Compensatory effort or dedifferentiation is always considered as an explanation of hyperconnectivity of brain regions (Cabeza et al., 2002; Grady et al., 2005; Guo et al., 2013; Su et al., 2015), which may be affected by inflammation process in the early state of schizophrenia. In that state (Anticevic et al., 2015), astrocytes could be activated by proinflammatory cytokines like interleukin-6, and consequently the metabolism and blood flow increased (Liberto et al., 2004). It is noteworthy that numerous thalamic nuclei comprise the thalamus, and there are topographically parallel pathways linking these anatomical segregated nuclei to different cortical regions within the thalamo-cortical circuits (Alexander et al., 1986; Haber, 2003; Woodward et al., 2012). Pergola and colleagues found that gray matter volume of the mediodorsal thalamic nucleus was associated with schizophrenia but state-related, while the left anterior and midline thalamic nuclei was the most important region associated with familial risk (Pergola et al., 2017). Decreased connectivity between the prefrontal cortex and dorsomedial/anterior thalamus was also observed in previous studies (Woodward et al., 2012). It is still unclear whether the increased GFC in the bilateral thalamus documented by our study pertains to specific thalamic nuclei and whether there are associations between functional and structural imaging findings relating to thalamus. In addition, age is a vital factor that should be considered from a neurodevelopmental perspective. According to Fair and colleagues, there were significant differences in the thalamo-cortical FC between children, adolescents, and adults (Fair et al., 2010).

The DMN, including brain regions such as the posterior cingulate cortex (PCC)/precuneus, medial prefrontal cortex (MPFC), angular gyrus (Andrews-Hanna et al., 2014), and parahippocampal gyrus (Raichle et al., 2001), is one of the most consistently disturbed resting-state networks in patients with schizophrenia. MPFC is involved in the regulation of emotional behavior and self-referential processing in the DMN (Chen et al., 2012; Yu et al., 2014) and the angular gyrus plays an important role in the language process, spatial cognition, and memory retrieval (Uddin et al., 2010). Therefore, disturbed DMN network connectivity may be linked to part of poor performance seen in patients with schizophrenia. Some researchers also found unaffected siblings having altered regional activity in certain brain areas of the DMN (Guo et al., 2014a,c). However, one study showed no marked FC difference within the DMN between patients with schizophrenia and controls (Wolf et al., 2011). The inconsistency may result from sample heterogeneity, sample size, and analysis methods. For patients with schizophrenia, illness duration and potential medication effects are also confounding factors. Consistent with our results, the ITG, important for emotional processing, social cognition (Guo et al., 2014b), and facial perception (Schultz et al., 2000), has been reported to have reduced FC in patients with schizophrenia as compared with healthy controls (Vercammen et al., 2010). Previous evidence also suggests that the impairment of temporal lobe and constituent parts in schizophrenia patients may be an important element in the emergence of auditory hallucinations and thought disorder (Seok et al., 2007). Intriguingly, one study suggested that deficit schizophrenia, a subgroup of patients with poorer treatment response and greater possibility to become chronicity compared to non-deficit schizophrenia, demonstrated structural and functional abnormalities in ITG (Yu et al., 2017).

In addition to the relatively small sample size, there are some limitations in this study. First, the scanning did not conduct again in the patients group after treatment. A longitudinal study is better to portray the continuous GFC alteration of brain networks in vulnerable people and patients with schizophrenia. Second, structural alterations, including gray matter and white matter, were not examined in this study. According to some researchers (Guo et al., 2012, 2015b), there were structural alterations in the gray matter and white matter in patients with schizophrenia. Hence, structural alterations underlying GFC remain unclear. However, the neuroimaging data of patients, siblings, and controls were preprocessed in the same way in order to minimize the effects caused by lack of structural examination in the present study. Finally, the study was based on resting-state fMRI without tasks involved. Therefore, it may restrict the generalizability of this study and the interpretation of underlying pathophysiology should be caution.

## Conclusion

In summary, this study is the first to explore voxel-wise brain-wide FC in first-episode drug-naive patients with schizophrenia and unaffected siblings. Dysconnectivity of the thalamo-cortical circuits may involve in the etiology of schizophrenia. Enhanced GFC in left SFG may serve as a potential endophenotype for schizophrenia.

## Data Availability

All datasets generated for this study are included in the manuscript and/or the supplementary files.

## Author Contributions

WG and JZ designed the study. WG, QS, ZZ, YD, YO, and PP collected the original imaging data. WG, FL, XS, JC, and JZ managed and analyzed the imaging data. WG and YD wrote the first draft of the manuscript. All the authors contributed to and approved the final manuscript.

## Conflict of Interest Statement

The authors declare that the research was conducted in the absence of any commercial or financial relationships that could be construed as a potential conflict of interest.
